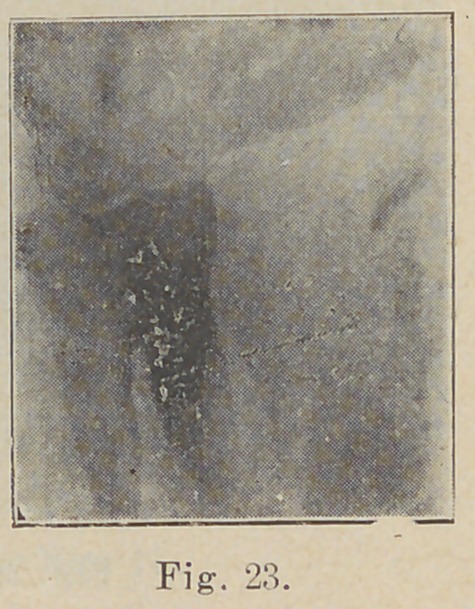# The Roentgen Rays with Associated Phenomena and Their Applications in Dentistry

**Published:** 1900-02-15

**Authors:** Weston A. Price

**Affiliations:** Cleveland, Ohio


### THE ROENTGEN RAYS WITH ASSOCIATED PHENOMENA AND THEIR APPLICATIONS IN DENTISTRY.

BY WESTON A. PRICE, D.D.S., M.D., CLEVELAND, OHIO.

The practical source from which we derive the Roentgen ray is from the point of impact, opposite a concave cathode, when a current of electricity is discharged through a gas in a vacuum tube. Usually this point is a plain platinum surface placed at from forty-five to sixty degrees to the direction of the cathode rays. These tubes may be excited by the discharge from an induction coil or a static machine or a Tesla coil or oscillator. As these devices have been quite fully described in current literature we will not stop to review them in detail, except to note the nature of a discharge through air.

Fig. 1.

The induction coil consists of a primary coil of a few turns of coarse wire surrounded by a secondary coil of a very great number of turns of very fine wire. When an interrupted or alternating current is passed through the primary, a current of very high voltage is induced in the secondary, demonstrating itself in discharges between the poles of the secondary. The phenomena are very beautiful in the dark, as you observe between these two suspended wires—lightning on a very small scale. The resistance of the atmosphere is so great that it takes probably several hundred thousand volts to discharge across that distance. When I place the discharging points closer together we get a continuous flame. When this discharge takes place through a tube partially exhausted the phenomena are very beautiful. I have a nice assortment of these tubes which I will now light up with the room darkened. You see both luminosity and fluorescence of the glass. At a certain degree of exhaustion the resistance to the passage of the current is very small. When I connect a lot of these Geissler tubes in series, you note, besides the gorgeous display, they all together, in which the current has to pass through many yards, do not offer as much resistance as a half inch of air gap. This, by the way, is the principle on which Tesla proposes to transmit an electric current from this country to Europe, without wires. At a certain altitude the atmosphere will be of this minimum resistance, and that stratum of air would constitute one path for the current and the earth the other.

Fig. 2.

I have some special tubes here also to demonstrate how the cathode rays produce fluorescence of certain salts and liquids. The display is very striking. Of the several methods for exciting the tube I prefer the induction coil, for which reasons can be brought out in the discussion. Next to the selection of the coil (I like the Ritchie very much), and almost of as great importance is the selection of an interrupter. Until recently the vibrator was the most commonly used break and probably is yet, though I think it is destined to be supplanted by the electrolytic breaks. The mechanical breaks are usually very noisy and the mercury breaks often troublesome. The Tesla oscillators are very noisy.

Fig. 3.

With the electrolytic break or interrupter, the capacity of the coil is greatly increased. It was discovered less than a year ago by Dr. Wehnelt in Europe, and this form bears his name. It consists of a fine platinum wire from sixteen to twenty gauge protruding from a glass tube in which it is sealed and a lead plate, both in a bath of dilute sulphuric acid. It will work on forty volts. I prefer using it on the one hundred and ten and have the acid solution so dilute that it serves at the same time as a rheostat. Great trouble has been experienced in making the platinum points adjustable, which is very desirable, and yet durable, owing to the heat and action of the acid. I have overcome this difficulty very successfully. The platinum wire is soldered with twenty-karat gold solder to a small brass bar of about the same diameter, and the whole placed inside a piece of clay pipe stem about eight inches in length (Fig. 1). The clay is not affected by either the heat or the cold, and while the apparatus is delicately adjustable it also is very durable; the pipe stem is adjustable through a rubber cork in the hard rubber top. I consider this form of interrupter very superior to any I know of. It has never failed to work in my hands. Its construction is very simple, as seen by this drawing (Fig. 1). Much could and should be said about the selection of tubes, but time will only permit of a word or two. Deal directly with a reliable firm and use pretty high vacuum tubes. I have sev-

Fig. 4.

eral forms. For a description of the most general forms I will refer you to Dr. Kell's admirable paper before the National Dental Association last August (*Dental Cosmos*, page 1014, 1899). I must speak here of one form, or rather addition, I have made which is different, and superior for its purpose to anything I have seen or heard of. It is for screening out all the luminous rays from the tube, while allowing the Roentgen rays to pass. It consists of a jacket of unvulcanized dental rubber. I use the red, which is opaque to the X-rays for all parts except where the rays are emitted, where I use a window of black rubber, through which the X-rays pass unobstructed. This tube is used for making fluoroscopic examinations in total darkness. It works to perfection and serves the double purpose of assisting to prevent danger of puncture. In general, I will say I like the Queen self-regulating tubes the best. My effort has been to reduce the time for getting good radiographs. Some of the best pictures I will show you this evening were taken in fifteen seconds through the dense jaws. This will be much reduced—to five seconds or less—for the average, in part by placing a piece of fluorescent screen, specially prepared, each in front and behind the celluloid X ray film, and part by using a tube of extra high current capacity—a new design of single focus alternating current tube of special merit made by Queen & Co.

Fig. 5.

Every class of case and age requires a tube of different condition and time of exposure; this requires a large assortment of tubes or adjustable tubes. This is the greatest factor in getting universal success, and should be made

Fig. 6.

the subject of a special paper. I may be able to give some hints when showing the practical cases. In our dental work the ordinary fluoroscope is practically of no use, except to show the condition of the tube. I have constructed some special screens which can be placed in the mouth and the shadow observed by a mouth mirror. This is only of service in a totally dark room, for which a tube with a jacket is designed. The same effect has been accomplished by others in a less convenient and simple way.

Fig. 7.

By far the most important way for us to use the X ray is to make a negative. For preparing the plate for this, many methods have been suggested. Some use entirely pieces of negative and wrap in black paper. Others use ordinary kodak film wrapped and sealed in black paper; some bind with lead or tin foil. To my mind no method that I have heard of approaches in convenience and simplicity the one I suggested and reported in my lecture before the Northern Ohio Dental Society last May. It consists simply in placing a large piece of a specially prepared X ray film which is quite stiff (Carbut's preferred) between two layers of unvulcanized black rubber, and allowing the rubber to touch around the edges where you dentists will best know how sure it will cling. This can now be cut through in any direction with a pair of shears and simply pressing the rubber together where you have cut seals a perfect joint. Of course, this is perfectly impervious to moisture and light, and at the same time offers almost no resistance to the X rays. I put a piece of Eastman's X-ray bromide paper in also with its face to that of the film, thereby getting a photo direct and also serving a double purpose in protecting the sensitive surface of the film. Occasionally it is an advantage to secure the plate with a special plate-holder, but very seldom do I think it desirable or necessary. The plates prepared in the way I speak of are so convenient you can bend a corner over anywhere, thereby making the plate any desired shape or size, and, of course, the rubber adheres to itself and holds it firmly. The plates can generally be held in place with a finger to get the best results. You will observe a number on each of the slides exhibited. This is done by placing little figures on the outside of the plate-holder. They are made of fine copper wire prepared in advance and stuck on gum labels. It is a great convenience for keeping records, without danger of getting negatives mixed. I have experimented with many opaque inks, but they are not satisfactory. I have a very complete system of keeping all the records of each case upon the envelope used as the negative preserver, which I will pass around. Note especially that the angles of the rays, the teeth and the plate to each other are kept in each case. This is very important and valuable since by it you can at any time determine the exact dimensions. Before proceeding with the practical

Fig. 8.

Fig. 9.

cases I want to advise any intending investors to get a small glass-top table on large, rubber-tire castors or wheels, for keeping your coil and accessories, which are few, upon. Also note that it has recently been shown that the time for exposure is in direct proportion to the distance, and not to the square of the distance, as has been generally thought.

We will now see some lantern views of practical cases, and one or two typical pictures will suffice in each of several classes of cases in which the Roentgen rays are specially well adapted for diagnosis. Unfortunately, a great deal of the detail and information is lost as compared with looking through the negative itself. Photos, even contact prints, lose about half their detail. In good negatives, even the cellular structure of the bone is distinctly shown. This is mostly or entirely lost on the screen or in the half-tone used for illustration, so you will have to make a great deal of allowance.

For the location of unerupted teeth it is simply perfection. Fig. 2 shows the condition of the superior arch of a boy at ten years of age. Two generations preceding, on his mother's side, had been lacking the superior laterals. She presented him to have steps taken to prevent the shedding of his deciduous laterals to make them permanent. You can scarcely imagine the joy of that mother, who herself suffers considerable disfigurement from this cause, when she saw the radiograph showing the development of these teeth that had been the object of her cherished but abandoned hope.

The next (Fig. 3) illustrates the condition in a case of delayed dentition of the bicuspids. The cuspid has been erupted for some time and the first deciduous molar shed by this eruption. This superior arch is exceedingly retracted. The radiograph shows that but one bicuspid has formed and it is thrown out of its course by about twenty degrees. It is absorbing the anterior buccal root of the first permanent molar. A course of treatment is quickly suggested.

In the next (Fig. 4) is a typical example of the appearance of cases where a permanent tooth fails to form and the deciduous is retained—in this case an inferior deciduous molar. A lady of about thirty years of age discovered that she still had a baby tooth and felt so ashamed of herself that she hurried to the office to have it extracted. The radiograph settles all hope of it ever having a successor.

I will now show you a wonderful picture demonstrating the very early calcification of the enamel of the permanent teeth (Fig. 5). It is taken of the superior arch of a baby boy of fourteen months, who has no deciduous teeth erupted yet in this arch. The radiograph shows not only the development of all the deciduous teeth, but also the commencement of calcification of the permanent centrals. But this is not all. While his father is lacking his permanent laterals we can see clearly in the negative, which was a ten-second exposure, even at this young age the crypts forming for his permanent laterals. I know of the joy of that boy's father at this information, for he is my boy.

Fig. 10.

An example of lost teeth is shown in the next (Fig. 6), which indicates the whereabouts of the missing bicuspids. There are very many cases of this class.

The next (Fig. 7) shows a lady's upper arch at about thirty years of age. When this picture was taken she was still waiting for her permanent teeth to erupt, but she need not wait longer for this lone second molar is all she is to have on that side. Five teeth are lacking on that side and three on the other.

In no class of cases is this means of diagnosis of more frequent service than for exploring the various deep pathological conditions. For example, the extent and location of abscesses, the direction and path of the fistula, and of very great importance, the most dependent point of the abscess. This picture (Fig. 8) shows the appearance of a blind abscess at the apex of an inferior incisor. It shows which tooth is affected and that no teeth in this vicinity have root fillings to suspect as imperfect.

Fig. 11.

In the next (Fig. 9) we have some information regarding a case of neuralgia of uncertain cause of years' standing undoubtedly caused, as proven later, by the blind abscess at the apex of the first superior bicuspid. The abscess was evidently caused by imperfect root-filling, which is clearly shown to only extend half way to the apex. On opening up the canal I found putrescence in the apical half of the canal. This negative shows beautifully the cellular structure of the bone.

Fig 12.

The next picture (Fig. 10) shows the same root filled to the apex. The blind abscess was drained through the buccal wall of the process.

Fig. 13.

These abscesses vary greatly in extent, and their exact extent is clearly shown in good radiographs, as for example in this picture (Fig. 11) which shows a blind abscess of considerable dimensions and of years' standing, during most of which time it has been almost continually under treatment, so the patient informed me, through the root-canal of the lateral where it had its inception. The lateral is the tooth nearest the figure 3. These numbers, you remember, are produced by placing metal figures, made of fine copper wire stuck on gum labels, upon the outside of the negative cover when the exposure is made, and are invaluable for keeping records correct and for identifying negatives. This picture shows beautifully the most dependent part of the abscess, which I have marked X, and the folly of trying to drain it through the root canal. On establishing free drainage at X, by drilling through the process and thoroughly sterilizing and cauterizing the pocket, a permanent and perfect cure was effected in a very few days.

Fig. 14.

In this next picture (Fig. 12) we have an exceedingly large abscess, and also one of long standing. It had a fistulæ beside the second bicuspid. The dentist sending the case for radiographing had labored faithfully, but with unsatisfactory results, to cure it. A crown on the lateral had been destroyed to examine the root-filling beyond it, and the second bicuspid had been extracted and replanted in search of exostosis. The radiographs, taken in sections, show the root of the lateral to be largely absorbed and a series of pockets difficult to drain between the roots of the various teeth. It also shows the abscess to extend beyond the anterior buccal root of the first molar.

Fig. 15.

The picture now before us (Fig. 13) shows a remarkable change of bone structure taking place around a superior central incisor, one that had, a couple of years previous, received a hard blow, since which time it has been constantly elongating, though it is very solid in its attachments. The negative shows clearly the old base for the apex, from which it has advanced about three-sixteenths of an inch. It is an interesting study in pathology. The bone, though forming an unusually firm attachment, is evidently less dense than normal, and slightly honey-combed in structure. The peridental membrane is almost obliterated.

Fig. 16.

In the next picture (Fig. 14) we have another blind abscess with its evident cause, viz., imperfect root-filling. The case was of thir-

Fig. 17.

Fig. 18.

teen years' standing and had received extended treatments. I amputated the tip of the root, without extraction, at the point where the root-filling ended, as shown by the radiograph (Fig. 14), and with splendid results. The next picture shows the same tooth after the root amputation (Fig. 15).

In the next picture (Fig. 16) we see how beautifully the bone has adhered around the stub of one of these amputated roots. This amputation was made in January, 1896. The patient claims it to be the strongest, solidest tooth she ever had.

Fig. 19.

In the next (Fig. 17) we see a broken broach protruding through the apex far into the tissue.

In this picture (Fig. 18) we see much of interest. The radiograph was taken to locate a piece of cambric needle which the patient had broken in the root while trying to relieve an abscess. It was thought to have been forced through into the tissue, as the apex was found open. It is easily

Fig. 20.

seen lodged in the root which was evidently bifurcated. This picture also shows beautifully the relation in this case of the teeth to the antrum. You will observe that the roots of the second molar penetrate nearly half way up through that cavity.

Fig. 21.

The value of Roentgen rays in orthodontia is almost beyond calculation, for it enables us to see just how ourforce is being expended.

Fig. 22.

Probably the greatest factor in that work is to move the roots as well as the crowns. In this picture (Fig. 19) we see an attempt to draw two teeth together, when alas! only their crowns have been tipped toward each other.

This next picture (Fig. 20) shows the commencement of a case where separation the width of a tooth (one having been recklessly extracted) is required. And in the next picture (Fig. 21) we see how well this has been accomplished. Unfortunately, time only permits of one example of each of a few classes of cases out of a great many.

Fig. 23.

The next (Fig. 22) shows beautifully the misfit of crowns and the depth of a pyorrhea pocket, supposed, marked X. The radiograph shows a perforation in the wall of the root of the first bicuspid through which cement has been forced into the tissue producing the suppuration and absorption, supposed to be a pyorrhea pocket.

In this next picture (Fig. 23) which is the last, we have an example of the service of the Roentgen rays in determining the location of third molars. This one is not erupted at all and lies just at right angles to its proper position, as shown by the second molar. It would certainly be a difficult one to extract if you did not know its position.

DISCUSSION.

DR. L. E. CUSTER: In regard to the manipulation of the tubes. When the X-ray was first discovered and put into use, tubes were used the vacuum of which could not be changed, so that a tube of very high vacuum would always remain at that, and it was impossible to lower it. We understand that as the vacuum becomes higher it is almost impossible to produce X-rays—the lower the vacuum the easier it is for the current to pass through.

In the later forms of tubes this has been overcome by the introduction into the tubes of potash, which on being heated partially vaporizes, and the vacuum is reduced to its proper degree. This is a very important thing in the work we have to do, for in going through a thick or deep tissue it is very necessary to have a high vacuum, while for the hand and the like it may be quite low. For fluoroscopic work it is quite important to have a high vacuum. You can, therefore, see how important it is to have a tube which can be regulated.

The method which Dr. Price has outlined in detail for procuring the skiagraph is certainly unique, and is to my mind the best thing that can be used.

Most of my experience has been with the Tesla coil, and I would say that I was led to invest in that upon the recommendation of Dr. Kells because of the very beautiful skiagraphs he exhibited. But he did not tell me that he was operating with an alternating current, so I placed my order according to his directions and they sent me one for the alternating current with which I was not supplied.

In order to operate on the direct current, a motor appliance is used which breaks the current and then alternates it. Owing to the large spark after about one hour's use, carbon brushes wear out, but by the substitution of copper for the carbon brushes, which would have a tangential contact, that trouble may be corrected.

I use two break-wheels instead of one, whereby the break of the current is more quickly made. The spark, which at first is three and one-half inches, afterward reaches about eight inches. It is solid, and as thick as a lead pencil, and there also extends out around this beautiful fluorescent sparks which are very beautiful. But when I came to use this, it melted the platinum in the tubes one after the other, yet the effect produced was something I have never seen in any X-ray appliance. The problem is to get a tube which will hold such a current and then we will have an instrument by which we could produce skiagraphs in seconds which now require several minutes On account of the difficulty in getting a tube I have lately ordered an induction coil such as you see here, except that it will give a fifteen-inch spark instead of a ten-inch spark.

The principle which underlies that of the induction coil is, that the longer the contact can be made, and the shorter the spark, the greater will be the induction.

The device which Dr. Price has used in conjunction with the interrupter is certainly a very useful one. If you have watched the history of the interrupter, you know it is a problem upon which the best electricians have been working, and I want to say that much credit is due Dr. Price for working out this difficulty in such a simple manner. It is the best thing I have seen or heard of.

Of course all different machines require different tubes. Those for use upon induction or static machines have one cathode disk and reflecting electrode, which throws the rays out at right angles. For the Tesla coil the tubes are called double focus tubes, because they have two cathodes and a double reflector which is not connected with the cathodes. They lack good definition because of forming two reflections. The results which have been obtained from them seem, especially in skiagraphic work, to be about as good as that obtained in other machines.

One advantage in the Tesla coil over the ordinary induction is the amount of time saved, which is about one half. This is not of great importance at this time, although formerly it was the cause of the burning we have all heard of.

I think there is scarcely one in the audience but will agree with me that a new field is opening to the profession, and that if we make use of the X-rays we will be much better prepared to serve our patients than heretofore.

It is not necessary for any one to get an expensive appliance. An ordinary induction coil to give a six or eight-inch spark, and two or three tubes, are about all the expense, and should not exceed $100.

Dr. Cassidy, Covington, Ky.: About twenty years ago one Crookes, an eminent English chemist, made a study of the properties of matter, and I think he may truly be said to be the discoverer of the whole business. He called it the "radiant matter." The discovery of the Roentgen rays was an accidental one, by one who was familiar with and informed of the radiant matter.

Crookes was somewhat tinctured with spiritualism, so when he discovered the radiant matter he immediately wrote a paper which was published in all the languages of the world, in which he stated that he had reached the borderland of spiritualism, and his writings at that time were away ahead of his days and equal to our present knowledge. When his paper first came out, it was regarded a little worse than a spirit. There are good and bad spirits, and I am almost forced to believe there are good and bad X-rays. A recent writer states that the X-ray can lie as irreligiously and with as little impunity as any other affidavit.

You all know what a lawyer can do when a question comes up; he can lie on one side and then on the other. A lawyer in Chicago placed his hand before an X-ray machine for the purpose of obtaining a picture, and the ghost picture came out with all parts in their normal condition. He then introduced this same hand before the machine, but bent the first joints a little, and the skiagraph in the second exposure showed the bones in a mashed condition, as though the fingers had been in a railroad accident; the parts were in an abnormal condition.

Another test was made. A bullet was placed under the clothing, outside of the body, and the picture showed it exactly against the spinal column. Of course this may be a trick with the density of the tube, or something we do not know about; but still, if this is the case, it is not reliable.

Dr. Butler: I have only a few words to offer in connection with the two excellent talks on this subject, the one last night and the other by Dr. Custer here this this afternoon. They have both simply showed me how useless it is for any dentist, however much he knows on this subject or how much skill he has, to cover all the ground of his dental practice, and do it in the best possible manner, and at the same time keep up in the X-ray work.

I think it is hardly worth while for us, as general practitioners in the profession, to try to master this subject, and the best thing to do is to turn over any case we may have into the hands of these specialists and let them help us out.

Dr. Taft: I want to refer to a case of which I have some knowledge, that occurred within the last five months. A lady had a fall and injured her shoulder, from which she suffered considerably. An effort was made to reduce a supposed dislocation. Success did not attend this effort, and she suffered right along. She went to see a surgeon and he suggested that an X-ray picture be made to determine the trouble. A skiagraph was made of the shoulder, the chest and that part of the body, with the hand and arm lying across the chest. The rays were applied and the picture obtained with apparently no effect to the patient; but within twenty-four hours a redness of the skin began to appear over all the surface which had been exposed to the rays, and within two or three days the whole surface looked as if it had been burned or scalded, and a blister raised over it, showing the same condition as any other kind of a burn. The cuticle came away with more or less discharge of serum and pus, and a very agonizing burn seemed to have been produced.

Within a week the hand used in holding the clothing also took on the same condition, first redness followed by formation of blister, raising of cuticle and discharge of serum, so it was impossible for the lady to dress herself. This condition remained for some time; the surgeons were nonplussed—had never seen anything like it—and knew not what to do only to wait.

After several weeks the skin had returned somewhat to its normal condition, although the hand was in an unusable condition for some time.

Now, the question is, Is that liable to occur? I have seen several cases of burns, but never anything as bad as this. Does it occur frequently, and is it hid from the public? If it is likely to occur, it comes up as a very serious and important question in connection with the use of X-rays. I would like to be enlightened on the subject.

Dr. Emminger: A number of you no doubt remember the case of Prof. Thomas, of the University, who last year was almost wholly incapacitated from his duties for some time, brought about by frequent experiments of the X-ray on his own body.

Dr. Price: Regarding the condition of the tubes. All tubes will tire, just as a machine or a person; you may work one for weeks and months, but it will finally become exhausted from overwork or too high vacuum. Then lay it away for two or three months, when it can be used with splendid results.

Another way of reducing vacuum in the tubes is to pass a very heavy discharge through them, such as the electrolytic interrupter will give. It will bring the vacuum away down. By heating the tubes you will also drive molecules of air that are condensed on the sides of the tube back to their vaporized condition. These are practical points which, though easily forgotten, are very important, so I call them to your attention.

Regarding the stories relating to the manipulation, by that Chicago lawyer, of his hands and the bullet in his back, and one thing and another, it is the biggest piece of bosh I have ever heard of. Any man of experience knows how to conduct the operation to get definite results, and if he sees such a plate he is bound to suspect it right away. If you shut one eye and look out on a lake at a boat, you can not judge how far away it is from the place where you are standing. Why is this? Perspective. Both eyes are necessary to focus it to a definite location. So we have this same principle in the use of the tubes. After getting the picture of the bullet from one plane, all you have to do is to turn the man half way around, and take another, and you know whether the bullet is in his vest pocket or in his back.

As far as burns are concerned, they are liable to happen through carelessness or accident, and will probably continue to happen, with less and less frequency, as long as the science continues to develop. It is now almost as positively known why and how they burn as you know why your finger will be burnt if you stick it into a lamp flame.

As long as you live up to the information of the day regarding the cause of this, it will not happen. The trouble occurs because of using tubes of too low vacuum, the rays of which are absorbed by the tissues instead of penetrating through them, and usually by very long exposures. In the case referred to by Dr. Taft, I could have told that they would not get a good picture, because they did not have rays of sufficient penetration. It takes a very high vacuum to get a picture through all that tissue. Fortunately for us, as dentists, we need tubes of very high penetration, because we have nothing to do with taking skiagraphs of soft tissues; it is for very dense tissues.

I will give you a little bit of practical experience. I have taken over two hundred and fifty skiagraphs, and I have never caused a particle of irritation except in one case, and in that one no inflammation. In that case I was looking for the cause of a bad case of neuralgia of very long standing, and made over twenty exposures within a very short space of time. By the way, the effect of these exposures is accumulative; if we make five to-day and five to-morrow, and the next day the same number, the effect will be the same as if we had taken all at one sitting. In our ordinary radiographs I never need make any exposures over three minutes, and usually ten seconds to one and a half minutes. The historic cases of X-ray burns were from long exposures—one half to two hours.

In the case above referred to we encountered the greatest density I have ever found, on account of this neuralgia. The bones were so abnormally dense that ordinary tubes, used with splendid success in other cases, gave no contrast between bone and roots of the teeth in her case. I wanted to get a tube of sufficient penetration to pass through the skull, so I sent for a large new tube for this case, and, as she was waiting to leave town, I did not stop to test it before using. I got it from the Edison people, supposed to be reliable.

After making several exposures and dismissing the patient, I tested the tube and found the spark equivalent very much lower than what our present knowledge knows to be required and consistent. There is no excuse for any manufacturer sending out a tube which will require less than a three or four-inch spark to start. This had one half inch, which was very, very low. Some weeks later I heard, through a friend, that some of her hair came out, but there was no indication of inflammation. The hair will grow in again. There has been no inflammation at all. I know very well why she was injured; it was because of the condition of that tube. I should have tested it before using, but as she was waiting to leave town I hurriedly put her in the chair and rushed the case along.

Dr. Custer: Did you use a screen between the patient and the tube?

Dr. Price: I did not use the screen. I use only very high penetration rays and short exposures, and, supposing the tube all right, I had no occasion to. If I had used a screen it would have prevented the trouble. An aluminum screen is very good, but I think a thin sheet of this red, unvulcanized rubber is the best. If the screen is metallic it is likely to spark to the tube, and there will be more danger of puncturing the tube. The red rubber is a non-conductor.

## Figures and Tables

**Fig. 1. f1:**
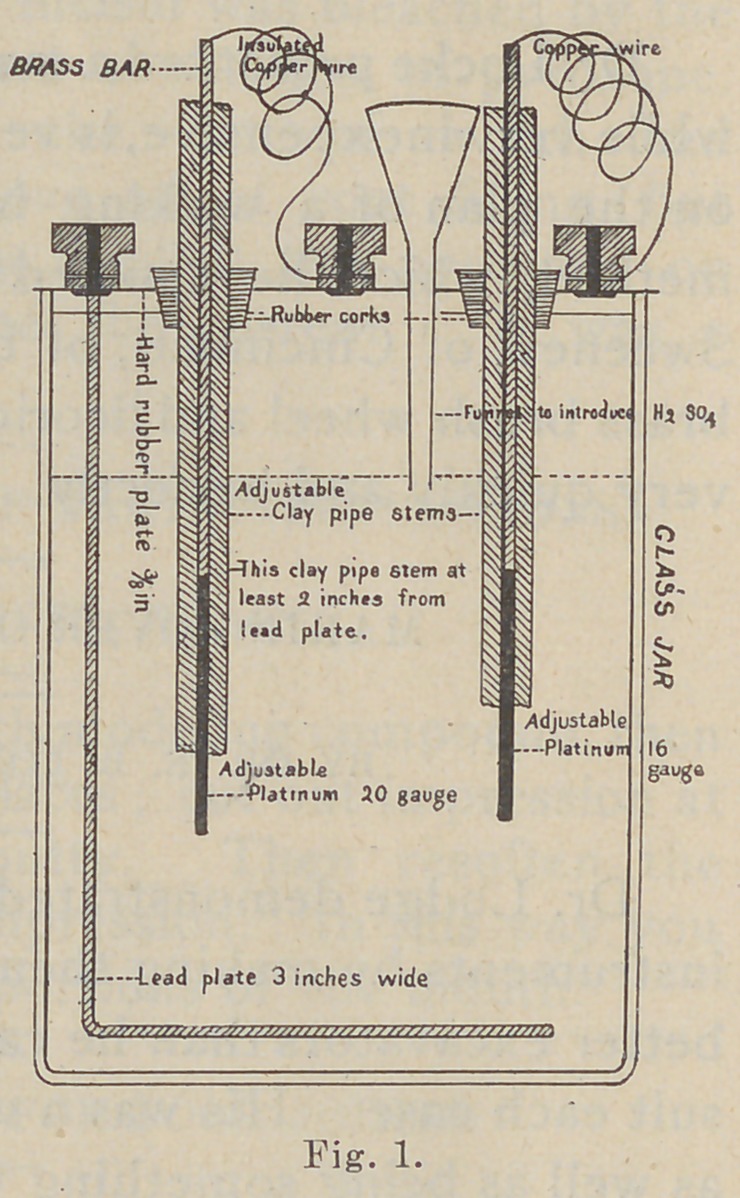


**Fig. 2. f2:**
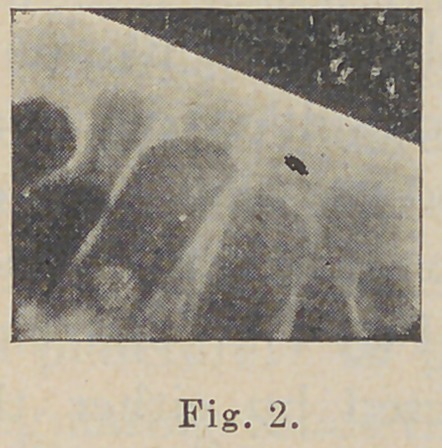


**Fig. 3. f3:**
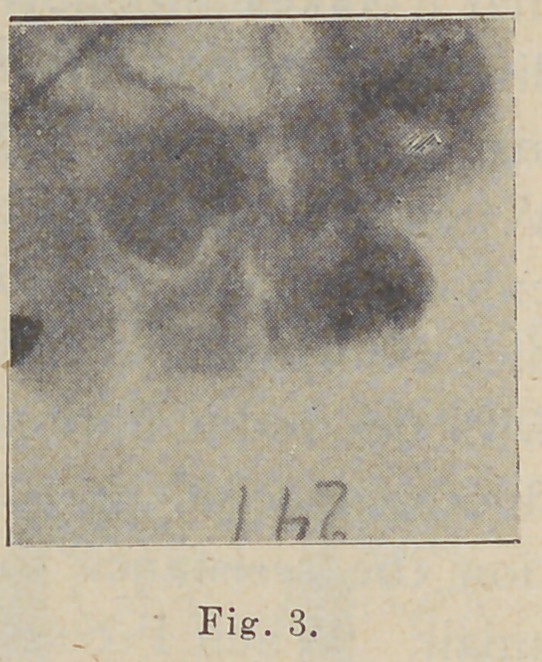


**Fig. 4. f4:**
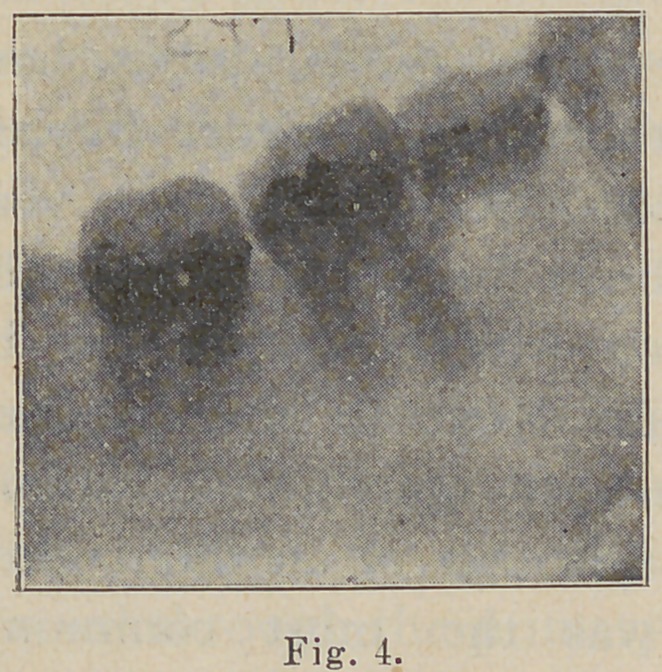


**Fig. 5. f5:**
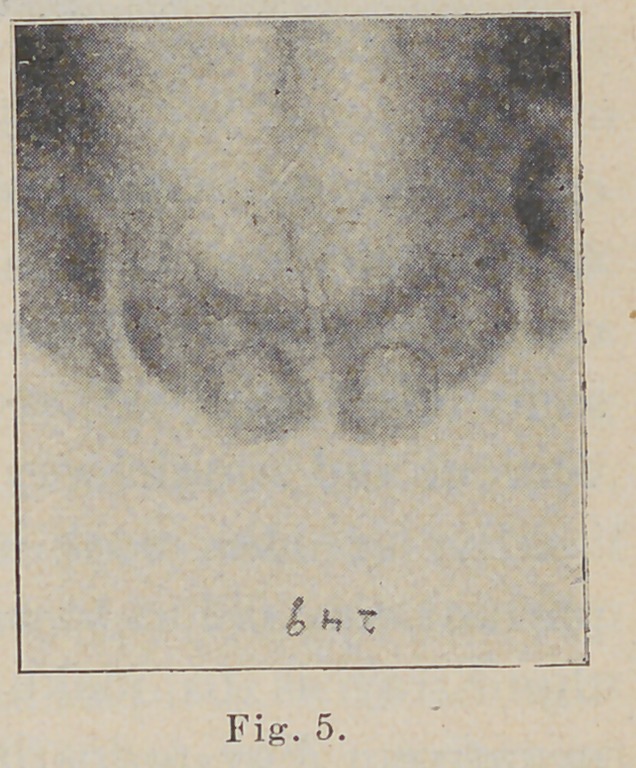


**Fig. 6. f6:**
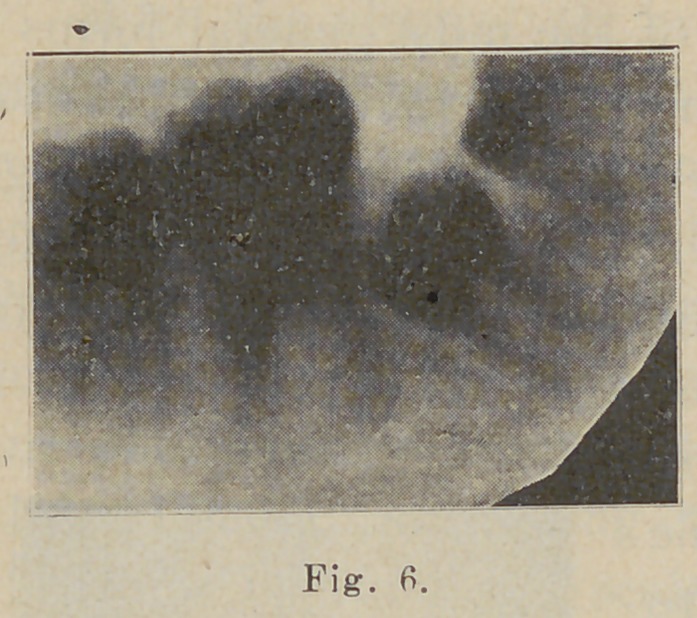


**Fig. 7. f7:**
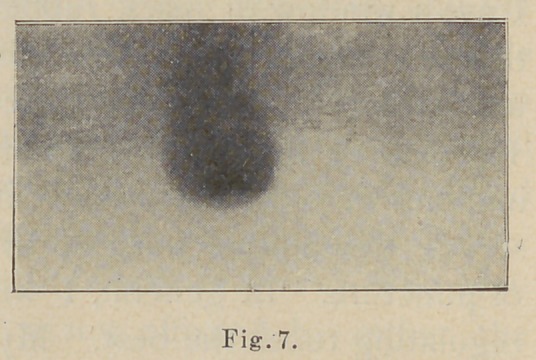


**Fig. 8. f8:**
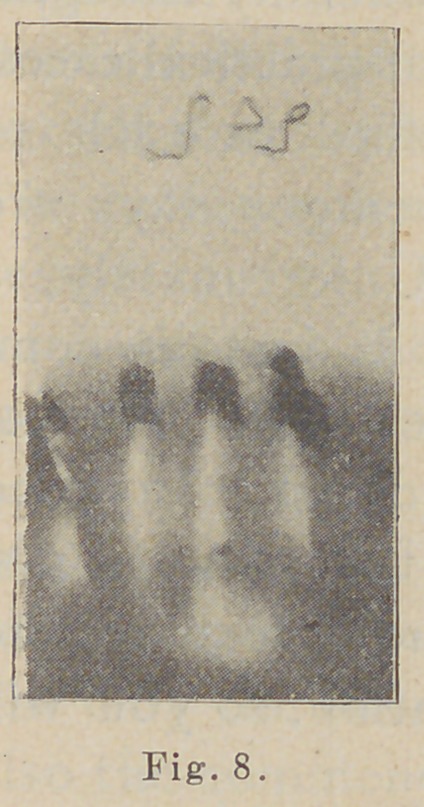


**Fig. 9. f9:**
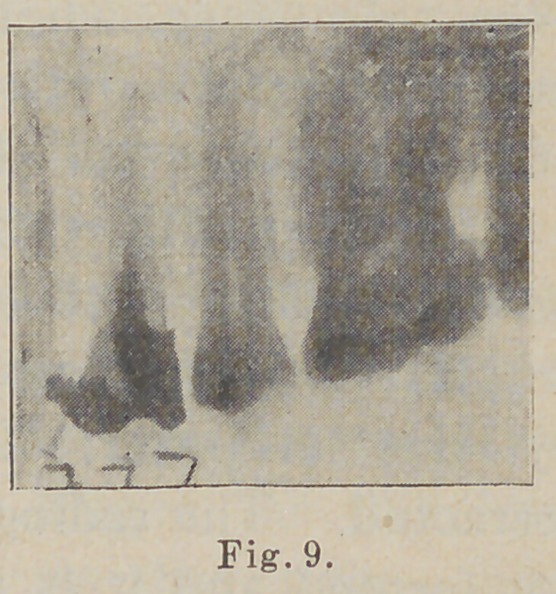


**Fig. 10. f10:**
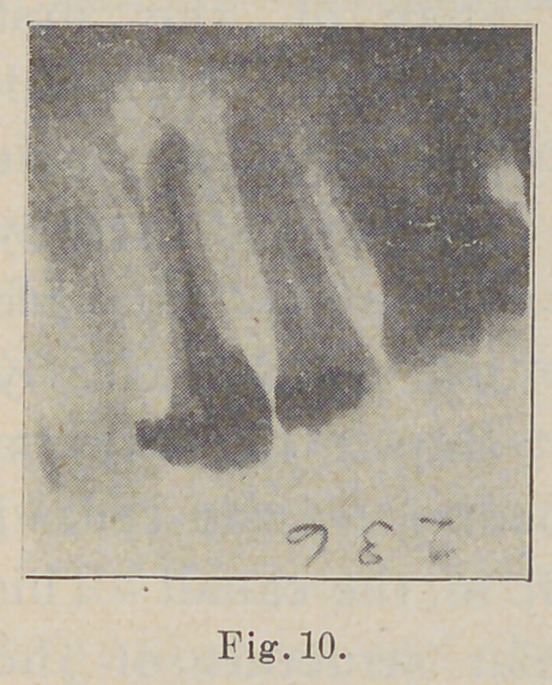


**Fig. 11. f11:**
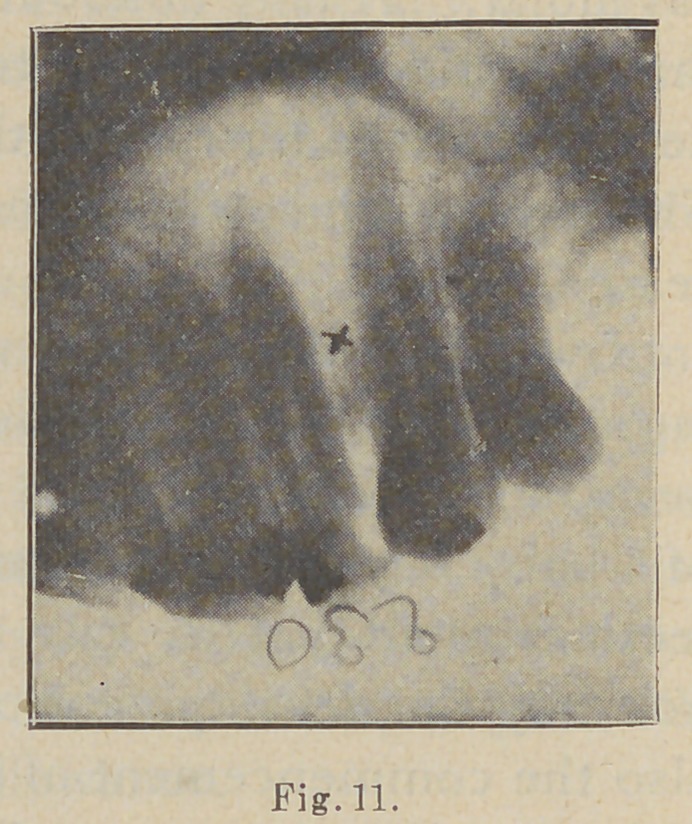


**Fig. 12. f12:**
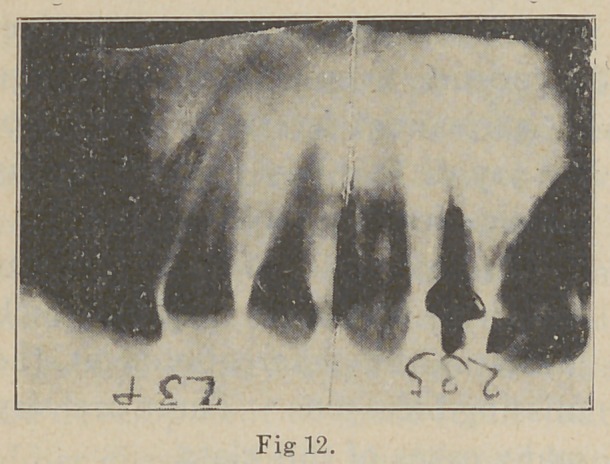


**Fig. 13. f13:**
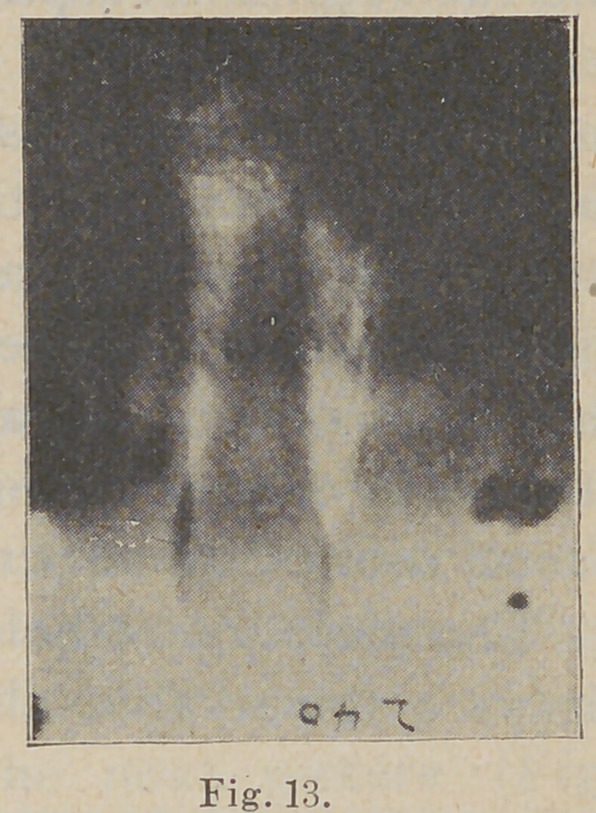


**Fig. 14. f14:**
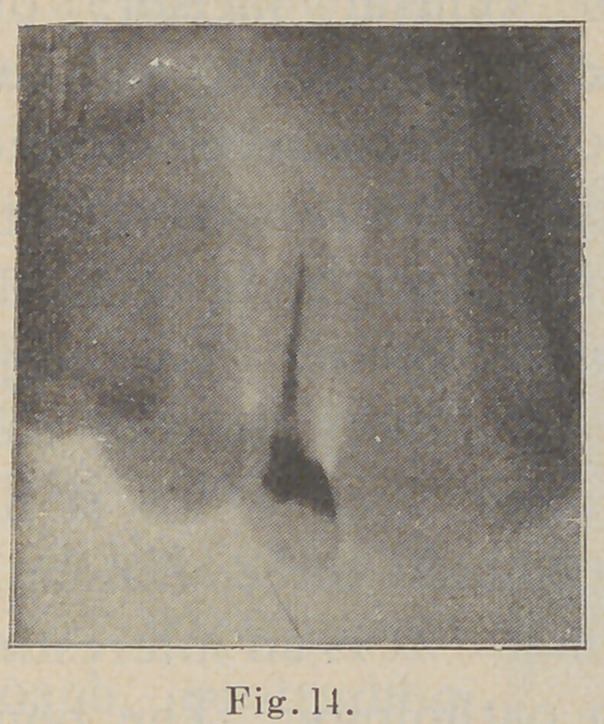


**Fig. 15. f15:**
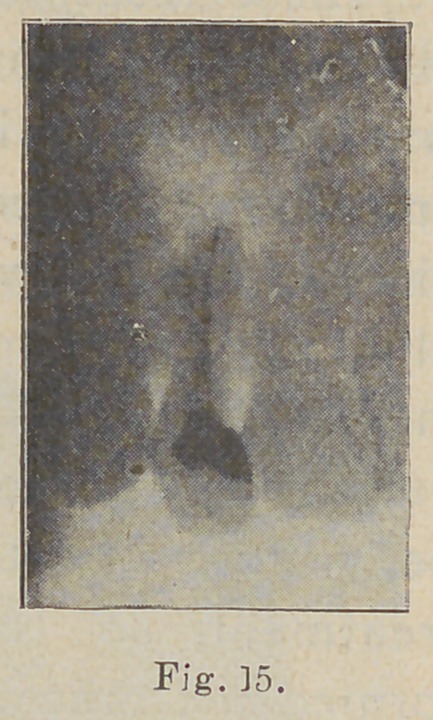


**Fig. 16. f16:**
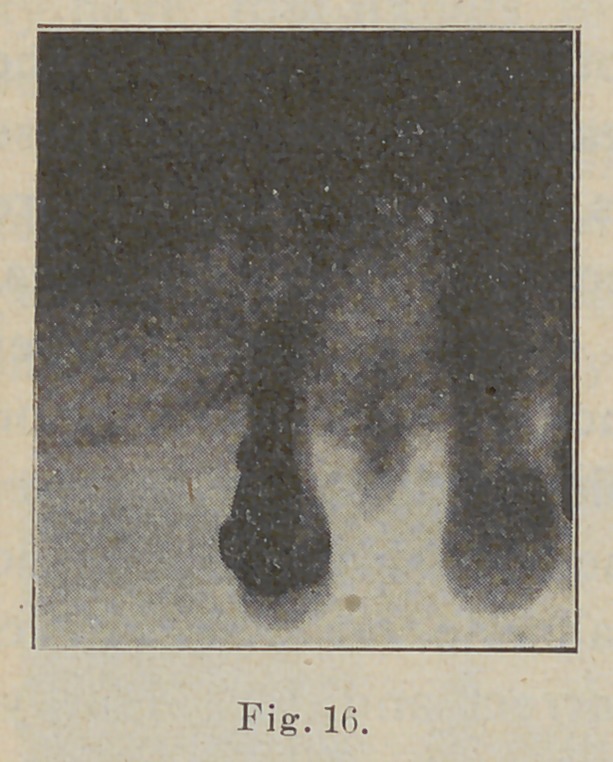


**Fig. 17. f17:**
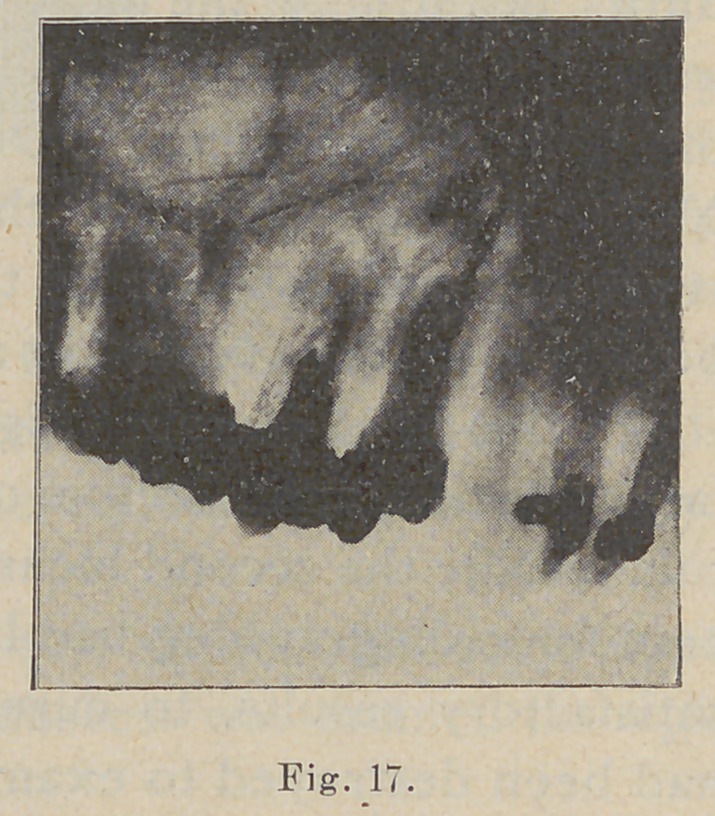


**Fig. 18. f18:**
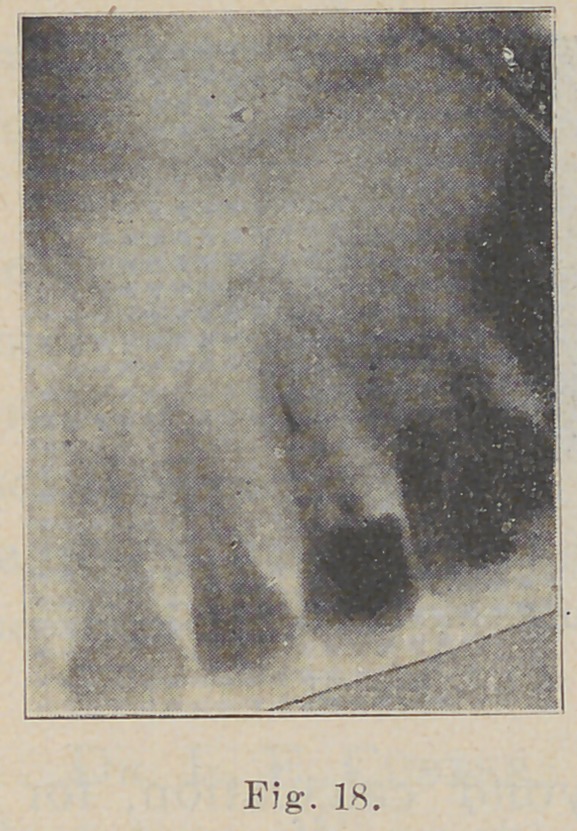


**Fig. 19. f19:**
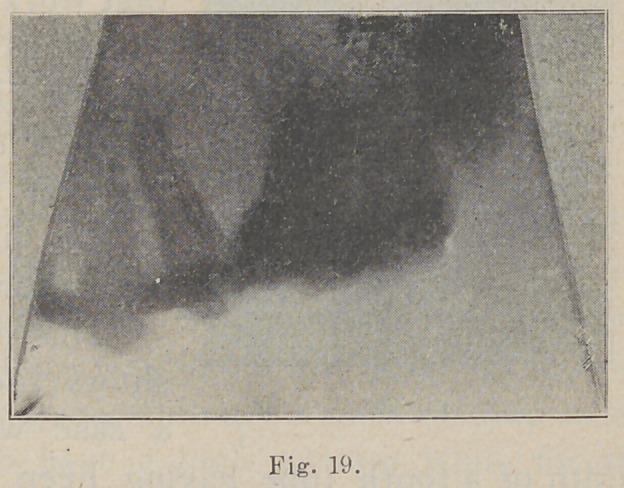


**Fig. 20. f20:**
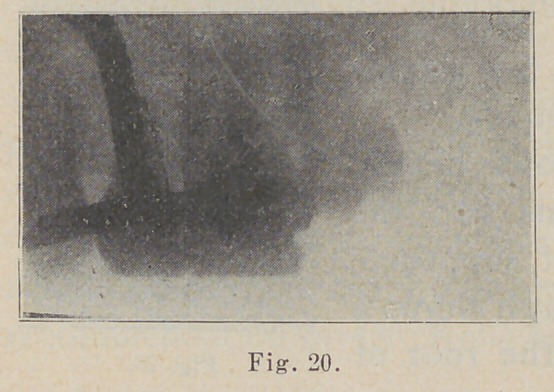


**Fig. 21. f21:**
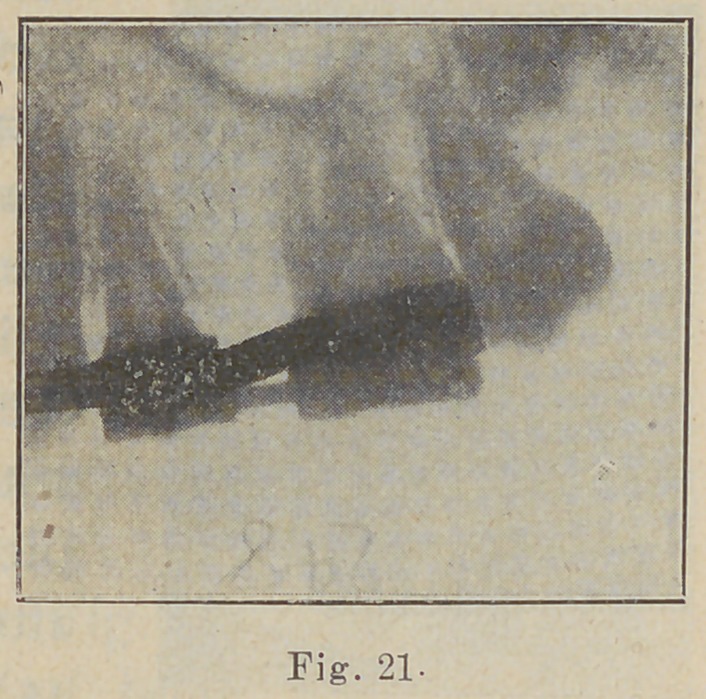


**Fig. 22. f22:**
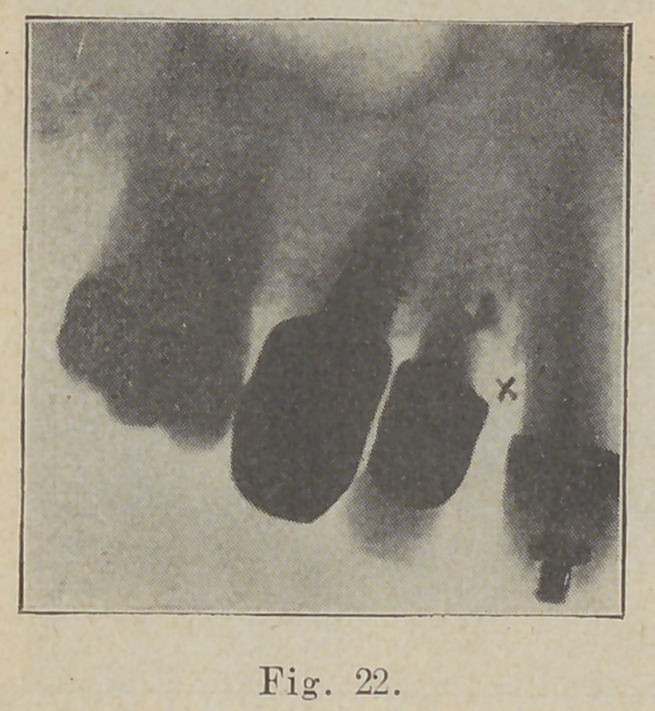


**Fig. 23. f23:**